# *Pseudomonas aeruginosa* Virulence Factors Support Voriconazole Effects on *Aspergillus fumigatus*

**DOI:** 10.3390/pathogens10050519

**Published:** 2021-04-26

**Authors:** Gabriele Sass, Pallabi Shrestha, David A. Stevens

**Affiliations:** 1 California Institute for Medical Research, San Jose, CA 95128, USA; Gabriele.Sass@cimr.org (G.S.); shrestha.pallabi@gmail.com (P.S.); 2 Division of Infectious Diseases and Geographic Medicine, Department of Medicine, Stanford University School of Medicine, Stanford, CA 94305, USA

**Keywords:** *Pseudomonas aeruginosa*, *Aspergillus fumigatus*, voriconazole, microbial interaction, cystic fibrosis, drug interaction, therapy

## Abstract

*Pseudomonas aeruginosa* and *Aspergillus fumigatus* are pathogens that are associated with deterioration of lung function, e.g., in persons with cystic fibrosis (CF). There is evidence that co-infections with these pathogens cause airway inflammation and aggravate pathology in CF lungs. Intermicrobial competition of *P. aeruginosa* and *A. fumigatus* has been described, but it is unknown how anti-fungal therapy is affected. The anti-fungal azole voriconazole (VCZ), supernatants of *P. aeruginosa* laboratory isolates PA14 or PAO1, or clinical isolate Pa10 independently inhibited biofilm metabolism of *A. fumigatus* isolates 10AF and AF13073. When VCZ and supernatants were combined at their IC_50_s, synergistic effects on *A. fumigatus* were found. Synergistic effects were no longer observed when *P. aeruginosa* supernatants were prepared in the presence of iron, or when *P. aeruginosa* mutants were lacking the ability to produce pyoverdine and pyochelin. Combination of pure *P. aeruginosa* products pyoverdine, pyochelin, and pyocyanin with VCZ showed synergistic anti-fungal effects. Combining VCZ with *P. aeruginosa* supernatants also improved its MIC and MFC against planktonic *A. fumigatus*. In summary, in the case of *P. aeruginosa*–*A. fumigatus* co-infections, it appeared that the *P. aeruginosa* co-infection facilitated therapy of the *Aspergillus*; lower concentrations of VCZ might be sufficient to control fungal growth.

## 1. Introduction

Persons with cystic fibrosis (CF), a hereditary disease caused by mutations in the genes specifying the cystic fibrosis transmembrane conductance regulator (CFTR), a chloride channel on epithelial cells [1,2,3], frequently suffer from bacterial, fungal, and viral co-morbidities of the lung that aggravate the course of disease. *P. aeruginosa*, members of the *B. cepacia* complex, *A. fumigatus*, respiratory syncytial virus, and influenza virus are prominent bacterial, fungal, and viral pathogens impairing lung function, especially when occurring in co-infections [4,5]. Therapies used during CF encompass drugs targeted against CF itself, encompassing two main classes of CFTR-targeting compounds: CFTR potentiators, increasing activity of CFTR on epithelial surfaces, and CFTR correctors, improving defective protein processing and trafficking [6], as well as drugs against each of the microbial infections, or co-infections. Microorganisms, and also drugs, interact, affect, and are affected by individual conditions in the lungs. *A.* *fumigatus* infections can include *Aspergillus* bronchitis, allergic bronchopulmonary aspergillosis (ABPA), and/or chronic progressive aspergillosis, and ultimately contribute to the need for lung transplantation [7]. In anatomically abnormal lungs, *Aspergillus* would persist as mycelial forms and generate biofilms. First-line treatment of ABPA is performed with oral corticosteroids [8,9], with concomitant side effects. Voriconazole (VCZ), itraconazole, posaconazole, and the anti-IgE antibody omalizumab might be alternatives [10,11,12]. Co-infections with *A. fumigatus* and *P. aeruginosa* trigger more severe outcomes than each mono-infection [13,14]. Interactions of *A. fumigatus* and *P. aeruginosa* have been studied for many years, with the majority of studies pointing towards an anti-fungal role of *P. aeruginosa*, interfering with fungal metabolism or growth via molecules such as its major siderophore pyoverdine [15], phenazines such as pyocyanin (5-N-methyl-1-hydroxyphenazine) [15,16,17], 1-hydroxyphenazine [15], phenazine-1-carboxamide [15], phenazine-1-carboxylic acid [15], and di-rhamnolipids [16]. We here aimed to illuminate interactions between bacterial molecules and anti-fungal therapy (use of VCZ) affecting *A. fumigatus* biofilm formation.

## 2. Results

### 2.1. Determination of IC_50_s for P. aeruginosa Strain PA14 Supernatants or VCZ against A. fumigatus 10AF Biofilm Formation

**Figure 1:**
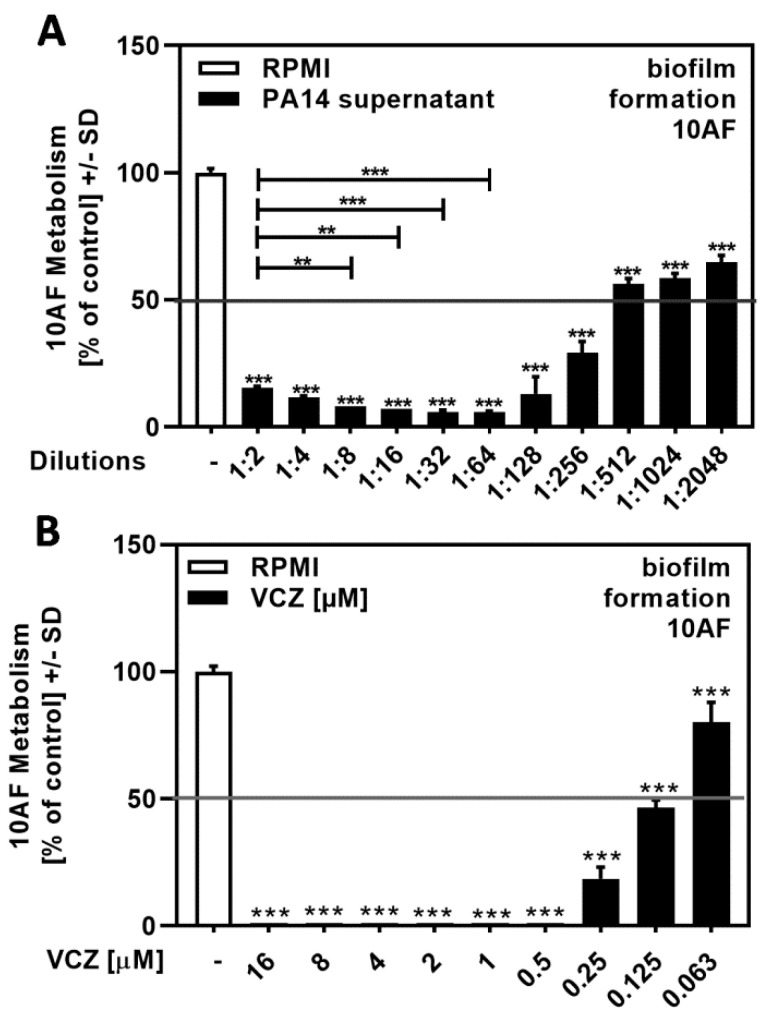
Dose–response curves showing IC_50_s for the antifungal effects of *P. aeruginosa* wild-type strain PA14 supernatant and VCZ dilutions against 10AF forming biofilm. PA14 wild-type bacteria (5 × 10^7^/mL in RPMI 1640 medium) supernatant in twofold serial dilutions (final concentrations: 1:2 to 1:2048) (**A**) or VCZ in twofold serial dilutions (final concentrations: 16 to 0.063 µM) (**B**) were added to 10AF conidia (10^5^ conidia/mL in RPMI 1640 medium). Assay plates were incubated at 37 °C overnight. Fungal metabolism was measured by XTT assay. Metabolism in the presence of RPMI alone was regarded as 100% and compared to each supernatant dilution. Statistical analysis: one-way ANOVA: two asterisks = *p* ≤ 0.01, three asterisks = *p* ≤ 0.001, respectively. Comparison: RPMI (white bar) vs. all other bars (black bars), or as indicated by the ends of the brackets.

Planktonic supernatants of *P. aeruginosa,* produced under limiting iron conditions in RPMI medium, contain the major siderophore pyoverdine, which inhibits *A. fumigatus* biofilm metabolism. Here, we determined the IC_50_ for PA14 supernatants by dilution in RPMI to be between 1:256 and 1:1024 (Figure 1A).

A previous study used VCZ concentrations between 0.125 and 1 µM for drug–drug interaction studies, with *A. fumigatus* forming biofilm metabolism as a readout [17]. Here, we determined the IC_50_ for VCZ against forming biofilm metabolism to be between 0.25 and 0.063 µM (Figure 1B).

### 2.2. PA14 Supernatant Supported VCZ Anti-Fungal Activity against 10AF

**Figure 2:**
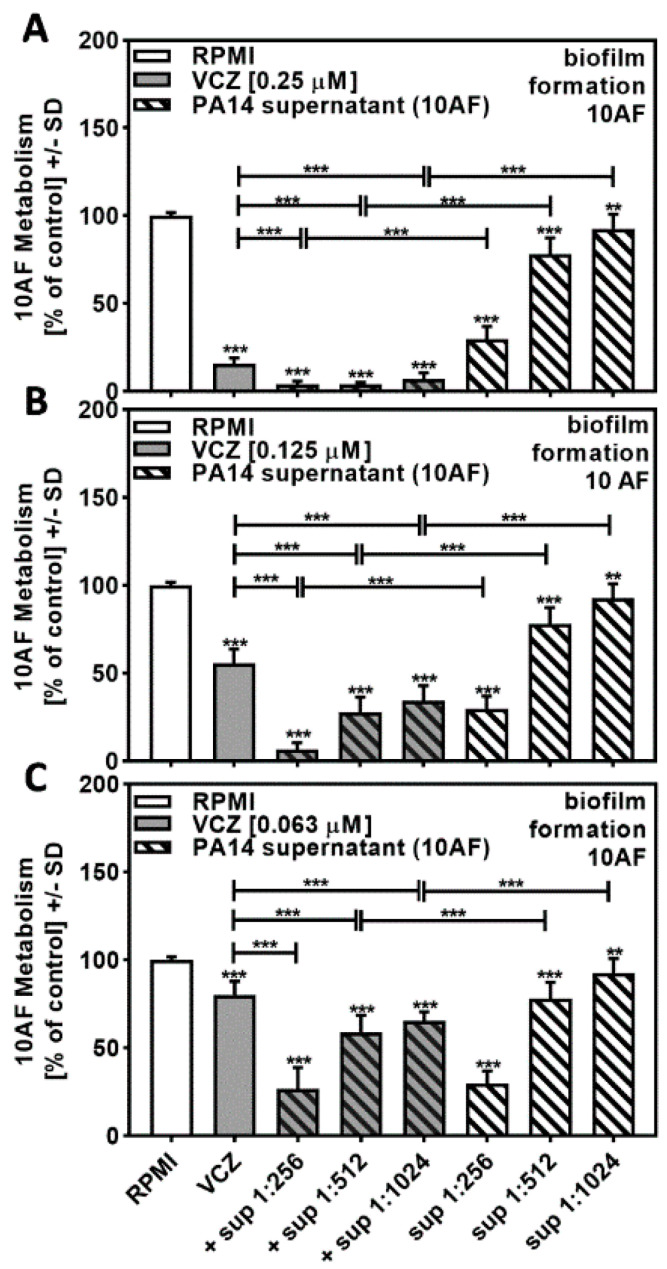
Combination of PA14 supernatant and VCZ significantly increased antifungal effects on forming 10AF biofilm. (**A**–**C**) PA14 wild-type bacteria (5 × 10^7^/mL in RPMI 1640 medium) supernatant was diluted to final concentrations of 1:256 to 1:1024 and combined with (**A**) VCZ 0.250 µM, (**B**) VCZ 0.125 µM, or (**C**) VCZ 0.063 µM to test their combined antifungal activities against 10AF forming biofilm (10^5^ conidia/mL in RPMI 1640 medium). Assay plates were incubated at 37 °C overnight. 10AF fungal metabolism was measured by XTT assay. Statistical analysis: metabolism in the presence of RPMI alone (white bar) was regarded as 100%, and compared by unpaired *t*-test to VCZ alone (gray bar) and PA14 supernatant dilutions alone (striped bars) and their combinations (gray striped bars). One-way ANOVA: VCZ vs. all VCZ combinations. Unpaired *t*-test for each supernatant dilution vs. its combination with VCZ: two asterisks = *p* ≤ 0.01, three asterisks = *p* ≤ 0.001.

Combining PA14 supernatant at concentrations close to its IC_50_ (twofold dilutions of 1:256 to 1:1024) with VCZ at concentrations close to its IC_50_ (0.25 to 0.063 µM) resulted in increased effects on forming 10AF metabolism at all combinations of PA14 supernatants with 0.25 µM (Figure 2A) and 0.125 µM VCZ (Figure 2B). At 0.063 µM VCZ (Figure 2C), only the two lower concentrations of supernatants increased the combined effects. Use of the BLISS Independence Model indicated that the interaction of PA14 supernatants with VCZ was mostly synergistic (Table 1 and Table S1).

Independent results calculated for the combinations of VCZ 0.250 and 0.063 µM with PA14 supernatant 1:256 were a result of strong individual component inhibition of 10AF metabolism. None of the combinations showed antagonistic effects.

### 2.3. Synergistic Interaction of VCZ with P. aeruginosa Supernatants Was Independent of the P. aeruginosa or A. fumigatus Strain Used

**Figure 3:**
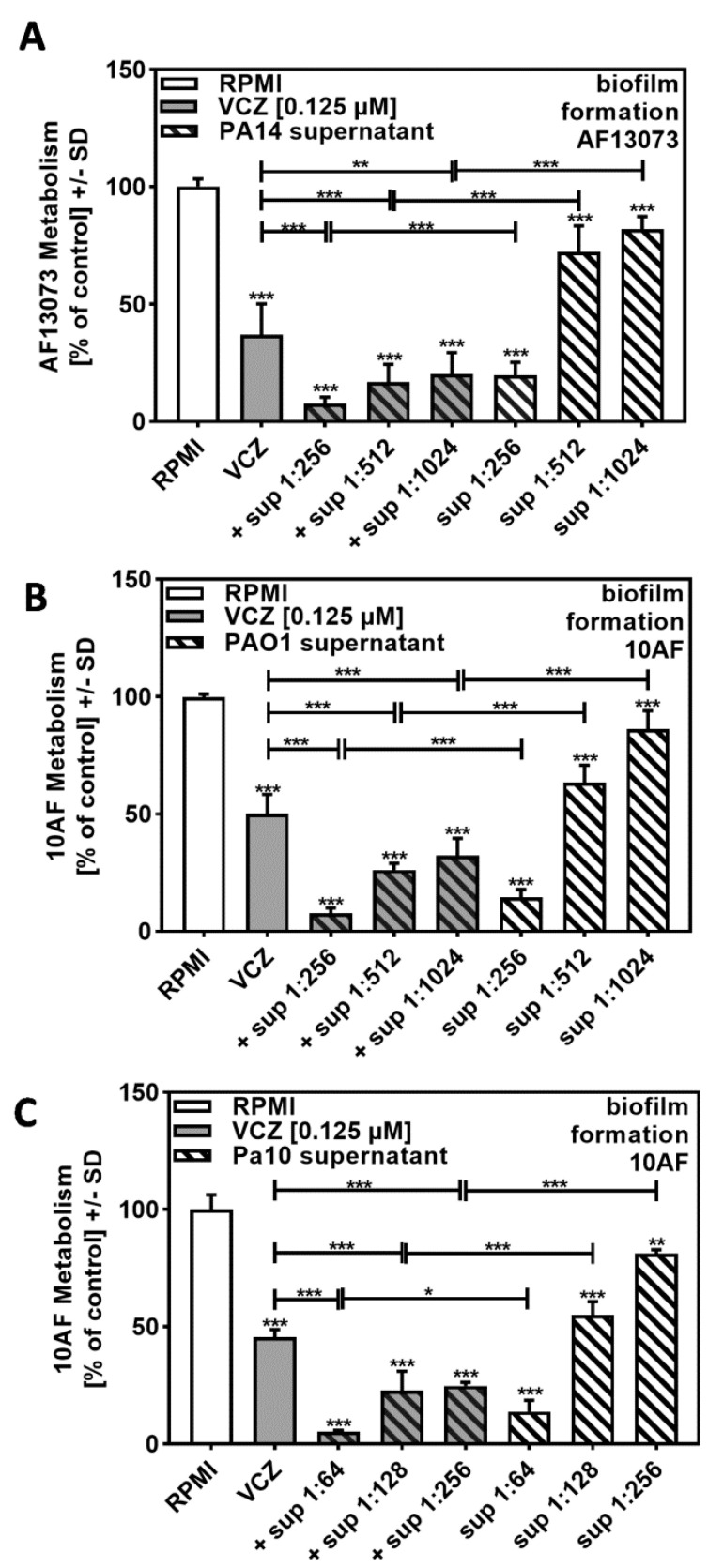
Increased anti-fungal effects by combinations of VCZ and *P. aeruginosa* supernatants were independent of the bacterial or fungal strains used. PA14 (**A**), PAO1 (**B**), or reference non-mucoid CF isolate Pa10 (**C**) bacteria (5 × 10^7^/mL in RPMI 1640 medium) supernatants were diluted to final concentrations of 1:256 to 1:1024 (**A**,**B**) or 1:64 to 1:256 (**C**), and combined with VCZ 0.125 µM to test their combined antifungal activities against AF13073 (**A**) or 10AF-forming biofilm (**B**,**C**) (10^5^ conidia/mL in RPMI 1640 medium). Assay plates were incubated at 37 °C overnight. Fungal metabolism was measured by XTT assay. Statistical analysis: metabolism in the presence of RPMI alone (white bar) was regarded as 100% and compared by unpaired *t*-test to VCZ alone (gray bar) and *P. aeruginosa* supernatant dilutions alone (striped bars) and their combinations (gray striped bars). One-way ANOVA: VCZ vs. all VCZ combinations. Unpaired *t*-test for each supernatant dilution vs. its combination with VCZ: One, two, and three asterisks: *p* ≤ 0.05, *p* ≤ 0.01, *p* ≤ 0.001, respectively.

In order to determine the biological range of VCZ–*P. aeruginosa*–*A. fumigatus* interaction, we studied another reference *A. fumigatus* strain, AF13073, and found similar effects (compare Figure 3A to Figure 2B).

We then studied supernatants of the widely used *P. aeruginosa* reference strain PAO1 and investigated combination of its supernatants with VCZ against 10AF forming biofilm metabolism. Our results showed similar effects as observed for the PA14 supernatant–VCZ interaction (compare Figure 3B to Figure 2B).

The BLISS Independence Model revealed overall synergistic and no antagonistic interactions between VCZ and bacterial supernatants. Table 2A,B was constructed for comparative purposes, whereas the calculation process and comparisons for interactions of bacterial supernatant with additional VCZ concentrations are provided in Table S2.

The most synergistic results were obtained when concentrations of bacterial supernatants were used, which by themselves had weak anti-fungal activity (1:1024 for PA14 and PAO1, 1:256 for Pa10).

Synergy of *P. aeruginosa* supernatants and VCZ against *A. fumigatus* forming biofilms is therefore not restricted to individual bacterial or fungal strains.

### 2.4. Iron Interfered with P. aeruginosa/VCZ Synergy

**Figure 4:**
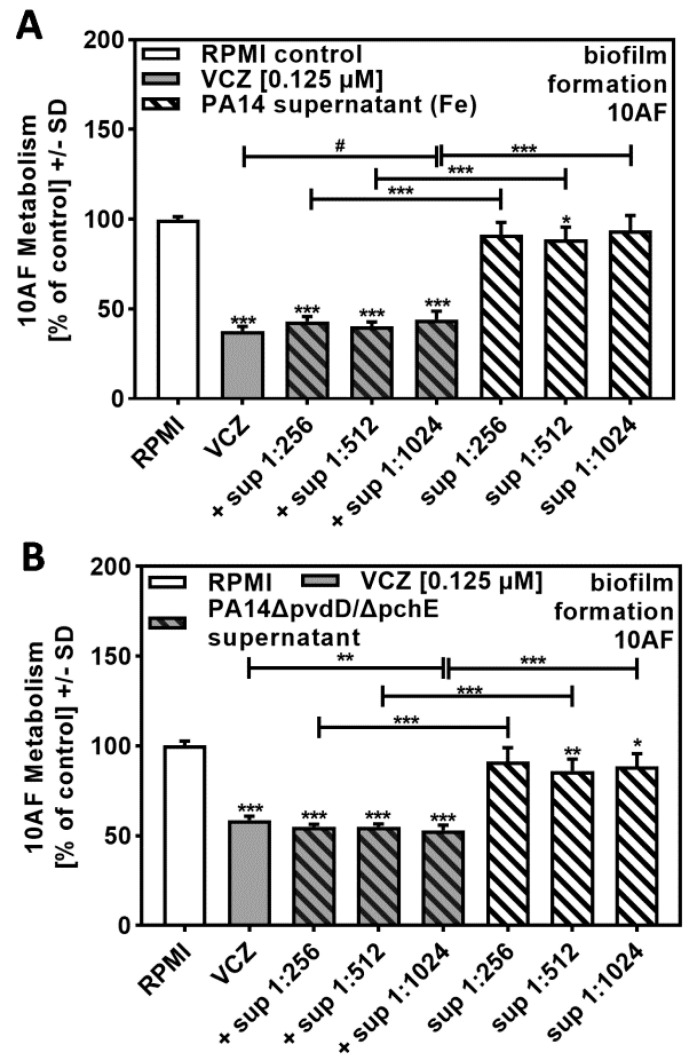
*P. aeruginosa* siderophores contributed to antifungal effects of VCZ and P. aeruginosa supernatants against *A. fumigatus* forming biofilm metabolism. PA14 supernatants (produced by 5 × 10^7^ bacteria/mL in RPMI 1640 medium, containing 50 µM ferric iron) (**A**), or PA14Δ*pvdD*/Δ*pchE* supernatants (produced by 5 × 10^7^ bacteria/mL in RPMI 1640 medium) (**B**) were combined with VCZ (0.125 µM) at dilutions of 1:256 to 1:1024. 10AF (10^5^ conidia/mL in RPMI 1640 medium) fungal metabolism was measured by XTT assay. Statistical analysis: metabolism in the presence of RPMI alone (white bar) was regarded as 100% and compared by unpaired *t*-test to VCZ alone (gray bar) and *P. aeruginosa* supernatant dilutions alone (striped bars) and their combinations (gray striped bars). One-way ANOVA: VCZ vs. all VCZ combinations. Unpaired *t*-test for each supernatant dilution vs. its combination with VCZ: one, two, and three asterisks: *p* ≤ 0.05, *p* ≤ 0.01, *p* ≤ 0.001, respectively. Asterisks indicate increased antifungal activities; pound signs indicate decreased antifungal activities. Separate experiments showed that added iron at the concentrations studied did not affect VCZ anti-fungal activity.

In order to determine molecules in *P. aeruginosa* supernatants that might contribute to synergistic reactions with VCZ against *A. fumigatus* forming biofilm metabolism, we prepared PA14 supernatants in the presence of added iron. Iron suppressed the production of the *P. aeruginosa* major siderophore pyoverdine, thereby decreasing anti-fungal activity. Figure 4A shows that bacterial supernatants, prepared in the presence of iron at a dilution of 1:256 or higher, no longer were anti-fungal (compare to Figure 1A) and were not able to act synergistically with VCZ (compare to Figure 2B).

BLISS Independence Model calculation revealed that interactions of supernatants, prepared in the presence of added iron and VCZ, were mostly antagonistic. Table 3, for comparative reasons using a VCZ concentration of 0.125 µM, showed antagonistic results throughout, whereas combinations of bacterial supernatants with additional VCZ concentrations also partially showed independent results (Table S3).

Supernatants of a PA14 mutant unable to produce the major siderophores pyoverdine and pyochelin (PA14Δ*pvdD*/Δ*pchE*) showed some anti-fungal activity of their own (Figure 4B). PA14Δ*pvdD*/Δ*pchE* supernatants in combination with VCZ showed no dose–response curve, and anti-fungal activities of the combinations were much weaker than observed for wild-type supernatants (compare Figure 4B to Figure 2B).

BLISS Independence Model calculations revealed that interactions were not synergistic, as observed for PA14 wild-type (compare Table 3 to Table 1). Further BLISS Independence Model calculations and interactions of bacterial supernatant with additional VCZ concentrations are provided in Table S3.

These data suggest that either pyoverdine or pyochelin, or a combination of both, could explain the positive interaction of *P. aeuginosa* supernatants with VCZ against *A. fumigatus* forming biofilm metabolism.

### 2.5. Pyoverdine Contributed to P. aeruginosa/VCZ Synergy

**Figure 5:**
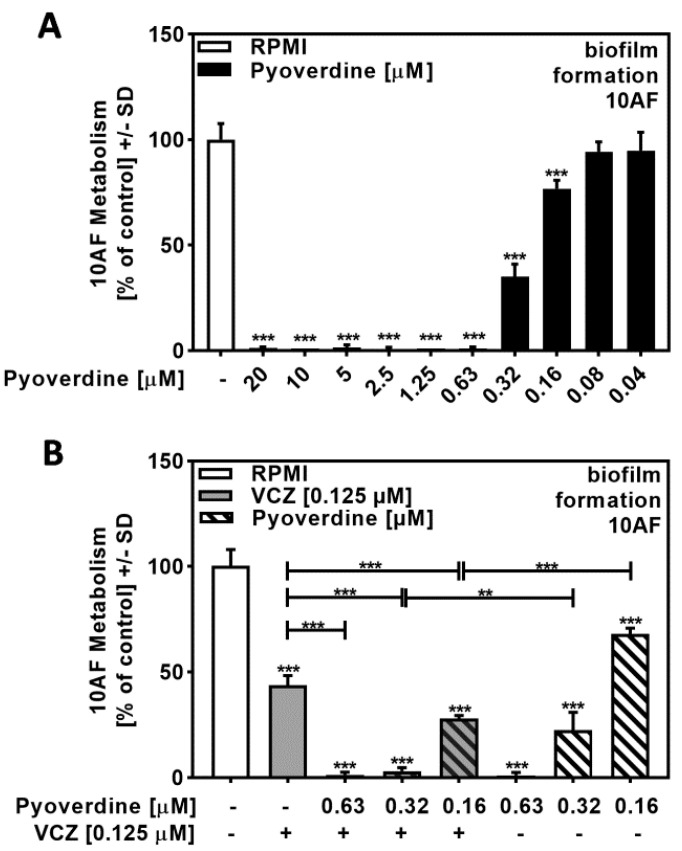
Pyoverdine significantly increased antifungal effects of VCZ against *A. fumigatus* forming biofilm metabolism. (**A**) Dilutions of pure pyoverdine in RPMI (20 to 0.04 µM) were tested for activity against forming 10AF (10^5^ conidia/mL in RPMI 1640 medium) biofilm metabolism. RPMI alone (white bar) was regarded as 100% and was compared to pyoverdine dilutions (black bars). Statistical analysis: one-way ANOVA: three asterisks: *p* ≤ 0.001. (**B**) Pure pyoverdine (0.063, 0.032, and 0.016 µM) was combined with VCZ (0.125 µM) and tested for combined antifungal activity against 10AF (10^5^ conidia/mL in RPMI 1640 medium) forming biofilm. 10AF fungal metabolism was measured by XTT assay. Statistical analysis: metabolism in the presence of RPMI alone (white bar) was regarded as 100% and compared by unpaired *t*-test to VCZ alone (gray bar), pyoverdine concentrations alone (striped bars), and their combinations (gray striped bars). One-way ANOVA: VCZ vs. all VCZ combinations. Unpaired *t*-test for each pyoverdine concentration vs. its combination with VCZ: two or three asterisks: *p* ≤ 0.01, *p* ≤ 0.001, respectively.

A dose–response study with pure pyoverdine indicated its IC_50_ to be approximately 0.32 to 0.16 µM (Figure 5A). Combining pure pyoverdine with VCZ revealed synergy against forming 10AF biofilm metabolism (Figure 5B, Table S4). Pyoverdine therefore enhanced VCZ anti-fungal activity.

### 2.6. Pyochelin Contributed to P. aeruginosa/VCZ Synergy

**Figure 6:**
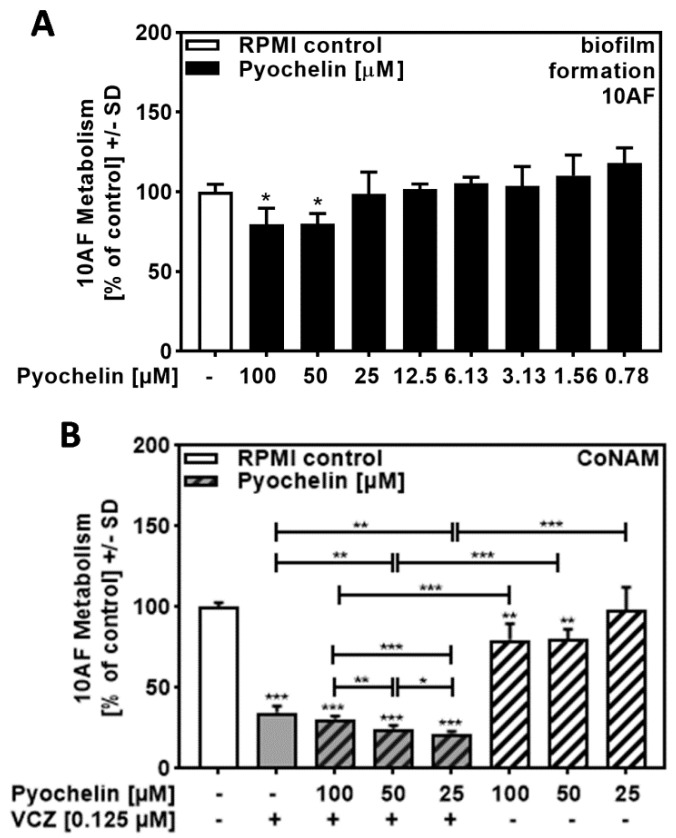
Pyochelin significantly increased anti-fungal effects of VCZ against *A. fumigatus* forming biofilm metabolism. (**A**) Dilutions of pure pyochelin in RPMI (100 to 0.78 µM) were tested for anti-fungal activity using forming 10AF (10^5^ conidia/mL in RPMI 1640 medium) biofilm assays. RPMI alone (white bar) was regarded as 100% and compared to all pyoverdine dilutions (black bars). Statistical analysis: one-way ANOVA: one asterisk: *p* ≤ 0.05. (**B**) Pure pyochelin (100, 50, and 25 µM) was combined with VCZ (0.125 µM) to test for 10AF (10^5^ conidia/mL in RPMI 1640 medium) forming biofilm metabolism. 10AF metabolism was measured by XTT assay. Statistical analysis: metabolism in the presence of RPMI alone (white bar) was regarded as 100% and compared by unpaired *t*-test to VCZ alone (gray bar), pyochelin concentrations alone (striped bars), and their combinations (gray striped bars). One-way ANOVA: VCZ vs. all VCZ combinations. Unpaired *t*-test for each pyochelin concentration vs. its combination with VCZ: one, two, and three asterisks: *p* ≤ 0.05, *p* ≤ 0.01, *p* ≤ 0.001, respectively.

Performing the same experiments as described in Figure 5, this time using pure pyochelin, we found no or weak anti-fungal activity for concentrations up to 100 µM (Figure 6A) but synergy of 25 to 100 µM pyochelin with VCZ (Figure 6B, Table S5). It is curious that lower concentrations of pyochelin in 6B had stronger effects in combination with VCZ compared to higher concentrations.

### 2.7. Pyocyanin Contributed to P. aeruginosa/VCZ Synergy

**Figure 7:**
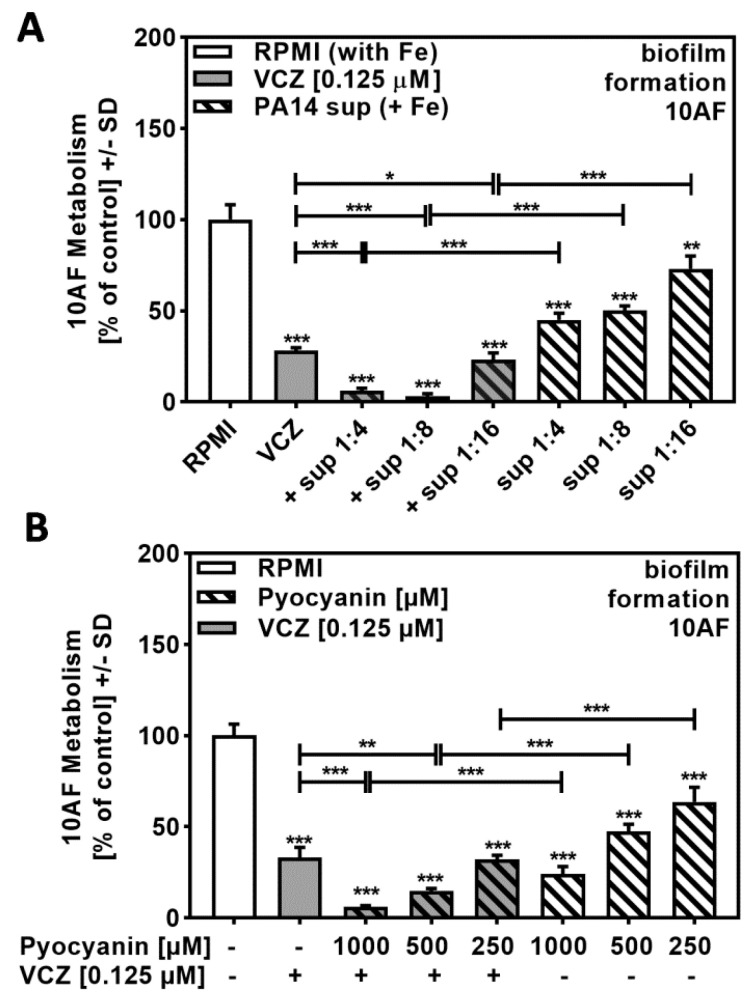
Phenazines/pyocyanin significantly increased anti-fungal effects of VCZ against *A. fumigatus* forming biofilm metabolism. (**A**) PA14 supernatant (produced by 5 × 10^7^ bacteria/mL in RPMI 1640 medium, containing 50 µM ferric iron, FeCl_3_) was combined with VCZ (0.125 µM) at dilutions of 1:4 to 1: 16. 10AF (10^5^ conidia/mL in RPMI 1640 medium) fungal metabolism was measured by XTT assay. Statistical analysis: metabolism in the presence of RPMI alone (white bar) was regarded as 100% and compared by unpaired *t*-test to VCZ alone (gray bar) and *P. aeruginosa* supernatant dilutions alone (striped bars) and their combinations (gray striped bars). One-way ANOVA: VCZ vs. all VCZ combinations. Unpaired *t*-test for each supernatant dilution vs. its combination with VCZ: One, two, or three asterisks: *p* ≤ 0.05, *p* ≤ 0.01, *p* ≤ 0.001, respectively. Separate experiments showed that added iron at the concentrations studied did not affect VCZ anti-fungal activity. (**B**) Pure pyocyanin (1000, 500, and 250 µM) was combined with VCZ (0.125 µM) to test their combined anti-fungal effects against 10AF (10^5^ conidia/mL in RPMI 1640 medium) forming biofilm metabolism. 10AF fungal metabolism was measured by XTT assay. Statistical analysis: metabolism in the presence of RPMI alone (white bar) was regarded as 100% and compared by unpaired *t*-test to VCZ alone (gray bar), pyocyanin concentrations alone (striped bars), and their combinations (gray striped bars). One-way ANOVA: VCZ vs. all VCZ combinations. Unpaired *t*-test for each pyocyanin concentration vs. its combination with VCZ: two or three asterisks: *p* ≤ 0.01, *p* ≤ 0.001, respectively.

Figure 4A revealed that high dilutions of iron-rich Pa supernatants (1:256 to 1:1024) did not act synergistically with VCZ, and Figure 5B and Figure 6B confirm the contribution of pyoverdine and pyochelin, respectively. Under non-limiting iron conditions, Pa not only represses pyoverdine production but also increases production of pyocyanin and other phenazines, which then exert anti-fungal activity. As iron-induced molecules might be diluted to ineffective concentrations when using 1:256 and higher dilutions of bacterial supernatants, we repeated the experiment shown in Figure 4A that involved high dilutions of iron-rich Pa supernatants combined with VCZ by using lower dilutions of PA14 supernatants that contain higher amounts of pyocyanin/phenazines. Our results showed decreased anti-fungal activity of iron-rich supernatants at dilutions of 1:4 to 1:8 in comparison to iron-limited supernatants (compare Figure 7A to Figure 1A), as well as increased anti-fungal activity in combination with VCZ (Figure 7A, Table S6).

As these supernatants do not contain pyoverdine (as verified by missing blue fluorescence under UV light [18]), but contain anti-fungal phenazines, we then tested for combined anti-fungal effects of pure pyocyanin, a phenazine induced by iron, and VCZ, and found positive interaction (Figure 7B, Table S6).

In summary, VCZ anti-fungal activity is supported by pyoverdine, pyochelin, and pyocyanin, depending on the milieu being limited, or not limited, for iron.

### 2.8. PA14 Supernatant Supported Anti-Fungal Activity of VCZ against Planktonic A. fumigatus Growth

We tested combined anti-fungal effects against planktonic *A. fumigatus* growth. We found that the combination with *P. aeruginosa* supernatants improved the MIC and MFC of VCZ with strong synergistic effects (Table 4). This corroborates the findings with *A. fumigatus* biofilms.

## 3. Discussion

*A. fumigatus*–*P. aeruginosa* coinfections trigger more severe outcomes than each mono-infection [13,14]. This could be a result of inflammatory signals caused by intermicrobial competition. Several *P. aeruginosa* molecules have been identified that interfere with fungal metabolism or growth via molecules such as phenazines, e.g., pyocyanin, or di-rhamnolipids [16]. Using defined media and an array of *P. aeruginosa* mutants, we recently found that under low iron conditions, pyoverdine is the major anti-fungal *P. aeruginosa* product, inhibiting *A. fumigatus* metabolism and growth by binding and withholding ferric iron from the fungus. Under high iron conditions, *P. aeruginosa* no longer produces pyoverdine, and thus anti-fungal effects of phenazines become more prominent [19]. Lungs of persons with CF tend to have higher iron content than infected non-CF lungs or healthy lungs [20]. The iron content in infected lungs varies by compartments, with mucoid plugs being low in iron [21], whereas micro-hemorrhages and hemoptysis provide higher iron levels. We also could show that the *P. aeruginosa* product 3,4-dihydroxy-2-heptylquinoline (PQS) uniquely affects *A. fumigatus* metabolism in two ways, depending on the concentrations of iron present: inhibiting the fungus under low iron conditions while promoting fungal metabolism under high iron conditions [22].

Treatment of *A. fumigatus* infections of the lung with azoles is common [10,11]. Given that *P. aeruginosa* products inhibit *A. fumigatus* metabolism, it seemed feasible that some of those products interacted with anti-fungal therapy. In fact, we found synergistic anti-fungal activity between VCZ and *P. aeruginosa* supernatants that was mediated by e.g., pyoverdine and pyochelin under low iron conditions, and pyocyanin under high iron conditions. VCZ MIC and MFC improved when combined with *P. aeruginosa* supernatants. There are likely other *P. aeruginosa* products able to synergistically interact with azoles and maybe with other anti-fungal agents that we did not test here. Preliminary results (unpublished) indicate that products of other bacterial species, e.g., the *Burkholderia cepacia* complex, affect azoles as well.

Through using several *P. aeruginosa* and *A. fumigatus* strains, we are confident that synergy between VCZ and Pseudomonas supernatants is a general feature during co-infections. We included a non-mucoid CF isolate (Pa10) and found synergy here as well. We previously could show, in an iron-restricted liquid milieu, that clinical Pa isolates, derived from persons with CF, have stronger anti-fungal activity than isolates derived from non-CF infections; moreover, mucoid Pa isolates from CF patients were less antifungal than non-mucoid isolates from CF patients. It remains to be seen if non-mucoid CF isolates have stronger synergistic effects than mucoid CF isolates with azoles.

An interesting phenomenon we observed during our studies was that low-iron *P. aeruginosa* supernatants at some dilutions increased in anti-fungal activity compared to undiluted or less diluted supernatants (e.g., Figure 1A). This phenomenon could either be explained by an increased protective response by *A. fumigatus* to high amounts of pyoverdine, or by the presence of pro-fungal factors in bacterial supernatants that at high concentrations mask part of their anti-fungal effects. For its own protection, *A. fumigatus* releases its siderophores into its growth medium to secure iron and withhold iron from its competitors [23]. Preliminary results show that *A. fumigatus* strains lacking SidA production also show the phenomenon of *P. aeruginosa* supernatant decreased anti-fungal activity at high concentrations, making it more likely that there also are pro-fungal factors in bacterial supernatants.

Another interesting phenomenon was observed when the *P. aeruginosa* product pyochelin was combined with VCZ (Figure 6B). We observed synergistic effects that, contrary to effects observed for other *P. aeruginosa* products, increased with decreasing concentrations. We cannot yet explain this observation, but hypothesize that we might examine pro-fungal effects of pyochelin in this situation.

In bacterial co-infections, VCZ might be effective at lower doses compared to *A. fumigatus* mono-infections. This might allow for the use of lower doses of VCZ and help to avoid azole side effects. The presence of certain coinfections, or the treatment for some mono-infections, will alter the subsequent microbiome [24]; can alter the subsequent clinical course; and could make initiation of, and choice of, antifungal therapy more cogent. Studies of any interactions with antifungal therapy need increased attention in view of some reports indicating rising antifungal resistance in Af isolates [25,26,27,28,29].

Although bacterial virulence factors would be unlikely to be clinically useful as anti-fungal agents, we see a wide field for the discovery of new agents here but would like to caution the community to the fact that treating bacterial co-infections of aspergillosis might require an adjustment in the dose of the anti-fungal used for therapy, and that treatment of *Pseudomonas* might make therapy of *A. fumigatus* more difficult.

## 4. Materials and Methods

### 4.1. Materials

Pyocyanin, pyoverdine, pyochelin, ferric iron (FeCl_3_), 2,3-bis(2-methoxy-4-nitro-5-sulfophenyl)-2H-tetrazolium-5-carboxanilide inner salt (XTT), menadione, and RPMI 1640 medium were purchased from Sigma-Aldrich (St. Louis, MO, USA). Iron contents in RPMI 1640 medium were below the detection limit (<1 µM, measured by inductively coupled plasma optical emission spectroscopy; Paolo Visca, Rome, Italy, personal communication). Voriconazole was obtained from Pfizer, New York City. Stock was prepared in DMSO and was further diluted to test conditions in RPMI. DMSO concentration in our combination experiments was 0.01%. DMSO concentrations below 1% do not affect *A. fumigatus* biofilm metabolism, and thus did not require dedicated DMSO controls. Large batches of the reagents were prepared in aliquots and frozen, and a fresh aliquot was used in each experiment.

### 4.2. Strains and Isolates

All bacterial and fungal strains used in this study are provided in Table 5. The use of all microbes in our laboratory is approved by the CIMR Biological Use Committee (approval No. 001-03Yr.15).

### 4.3. P. aeruginosa Planktonic Supernatant Production and Dilution

*P. aeruginosa* supernatants were prepared as detailed previously [36]. Briefly, *P. aeruginosa* wild-type or mutant bacteria (5 × 10^7^ cells/mL) were incubated in RPMI 1640 medium (Sigma-Aldrich) with or without the addition of 50 μM FeCl_3_ at 37 °C and 100 rpm for 24 h. Bacterial cultures were centrifuged at 2000 rpm for 30 min at room temperature and filtered for sterility (0.22 μm). Supernatants were diluted in RPMI in 1:2 steps with final concentrations ranging from 1:2 to 1:2048.

### 4.4. Assay for Measurement of Aspergillus Forming Biofilm Metabolism

*A. fumigatus* conidia (10^5^/mL final concentration) were distributed into the wells of sterile flat-bottom 96-well culture plates at 50 µL/well. Bacterial supernatants or test substances and VCZ were combined in equal parts by volume (25 μL each) to the final concentrations indicated. Final volumes in wells during assays were 100 µL. RPMI 1640 medium served as the negative control. The assay plates were incubated at 37 °C overnight, and hyphae growth was verified by optical microscopy before performing XTT assays.

All assays were evaluated by XTT metabolic assay as detailed previously [36,37]. Briefly, 150 µL of an XTT/menadione mixture (150 µg/mL XTT, 30 µM menadione) were added to each test well and incubated at 37 °C for 1 h. Supernatants from each well were transferred to a fresh 96 well plate (100 µL) and assayed using a plate reader (Vmax, Molecular Devices, San Jose, CA, USA) at 490 nm.

### 4.5. Minimal Inhibitory Concentration (MIC), Minimal Fungicidal Concentration (MFC), Fractional Inhibitory, and Fungicidal Concentration Indexes (FICi and FFCi)

Fifty microliters of VCZ (drug A, range 0.09–45.8 µM, corresponding to 0.032–16 µg/mL) was distributed in rows and 50 µL of *P. aeruginosa* supernatant (drug B, range 1:10–1:2560) was distributed in the columns for the interaction. Nine hundred microliters of standardized inoculum were added to the tubes. Tubes were incubated for 48 h at 35 °C before reading of MICs. Fractional inhibitory concentration index (FICi) was determined by the equation: FICi = (MIC_A_ in combination/MIC_A_ tested alone) + (MIC_B_ in combination/MIC_B_ tested alone). The fractional fungicidal concentration index (FFCi) was calculated and interpreted in the same way as described for FICi. The assay was performed in duplicate.

Drug interactions were classified as strong synergism when FICi or FFCi < 0.5; weak synergism when 0.5 ≤ FICi or FFCi < 1; additive when 1 ≤ FICi or FFCi < 2; indifferent when FICi or FFCi = 2; and antagonistic when FICi or FFCi > 2 [38].

To determine the minimum fungicidal concentration (MFC), we plated 50 µL of each tube without visual growth on Sabouraud agar and incubated the mixture at 35 °C for 24 h. MFC was considered the minimal concentration of the drug resulting in killing ≥99% of the inoculum.

### 4.6. BLISS Independence Model for Analysis of Drug Combination Effects

Combined drug effects were also calculated using the BLISS Independence Model as described previously [39]. Briefly, if drugs A (VCZ) and B (*P. aeruginosa* supernatant) inhibit Y_a_ and Y_b_ percent of growth, respectively, their predicted combined effect (considering they work independently) is given by the following formula: Y^p^_ab_ = Y_a_ + Y_b_ − Y_a_Y_b_. The predicted combined effect is compared to the observed combined effect (anti-fungal activity by the drug combination in XTT assays). The result is interpreted as

- Observed > Predicted: Synergy
- Observed = Predicted: Independent (5% range of Y^p^_ab_)
- Observed < Predicted: Antagonism

(abbreviations: S = synergy, I = independence, A = antagonism, Y_a_ = inhibition of fungal metabolism by respective VCZ, Y_b_ = inhibition of fungal metabolism by respective Pa sup, Y^o^_ab_ = observed combined antifungal effect, Y^p^_ab_ = predicted combined antifungal effect).

### 4.7. Statistical Analysis

Results were analyzed using Student’s *t*-test if two groups were compared, and one-way ANOVA combined with a Tukey’s post-test for multiple comparisons. All data in this study are expressed as a mean ± SD. Data reported as the percent of control were compared using Student’s *t*-test after arcsin transformation of the proportions; these data are presented as the percentages. Each assay was performed with three to eight biological and technical replicates. Representative experiments are shown.

## Figures and Tables

**Table 1:** Bliss Independence Model (PA14 supernatant and VCZ combination effects against 10AF forming biofilm).

	VCZ 0.250	VCZ 0.125	VCZ 0.063
Pa sup 1:256	I	S	I
Pa sup 1:512	S	S	S
Pa sup 1:1024	S	S	S

(S = synergy, I = independence).

**Table 2:** Bliss Independence Model (PA14 supernatant and VCZ combined effect against AF13073 forming biofilm and *P. aeruginosa*, PAO1 and Pa10, supernatant combined effects with VCZ against 10AF forming biofilm).

A	PA14 Sup (10AF)	PA14 Sup (AF13073)	PAO1 Sup (10AF)
Pa sup 1:256	S	I	I
Pa sup 1:512	S	S	S
Pa sup 1:1024	S	S	S
	VCZ 0.125	VCZ 0.125	VCZ 0.125
**B**	**Pa10 Sup** **(10AF)**		
Pa sup 1:256	I		
Pa sup 1:512	I		
Pa sup 1:1024	S		
	VCZ 0.125		

(S = synergy, I = independence).

**Table 3:** Bliss Independence Model (PA14 supernatant in RPMI with iron, and PA14Δ*pvdD*/Δ*pchE* supernatant combination effects with VCZ against 10AF forming biofilm).

	PA14 Sup (In RPMI with Iron)	PA14Δ*pvdD*/Δ*pchE*
Pa sup 1:256	A	I
Pa sup 1:512	A	A
Pa sup 1:1024	A	I
	VCZ 0.125	VCZ 0.125

(I = independence, A = antagonism).

**Table 4:** MIC, MFC, FICi, and FFCi values for PA14 supernatants, VCZ, as well as combinations of both against planktonic 10AF growth.

	MIC	MFC	FICi	FFCi
PA14 supernatant	1:32	No MFC	-	-
VCZ	0.5 µM	16 µM	-	-
Combination	1:32 + 0.125 µM	1:256 + 8 µM	0.28	0.5

**Table 5:** Strains and isolates used in this study.

Organism	Isolate	Description	ATCC	References
*Aspergillus fumigatus*	10AF	Virulent patient isolate	90,240	[30,31]
*Aspergillus fumigatus*	AF13073		13,073	
*Pseudomonas aeruginosa*	PA14	Parental strain of all PA14 mutants studied		[32,33,34]
*Pseudomonas aeruginosa*	PAO1		15,692	[35]
*Pseudomonas aeruginosa*	Pa10	Reference non-mucoid CF isolate		[36]
*Pseudomonas aeruginosa*	PA14Δ*pvdD*/Δ*pchE*	Pyoverdine-pyochelin double siderophore mutant		

## Data Availability

Raw data for these studies are available from Gabriele.Sass@cimr.org.

## Supplementary Materials

The following are available online at https://www.mdpi.com/article/10.3390/pathogens10050519/s1, Table S1: Bliss Independence Model calculation: PA14 supernatant and VCZ (µM) combined effects against 10AF forming biofilm. Table S2: Bliss Independence Model calculation: PA14 supernatant and VCZ (µM) combined effects against AF13073 forming biofilm and *P. aeruginosa*, PAO1 and Pa10, supernatants, combined with VCZ against 10AF forming biofilm. Table S3: Bliss Independence Model calculation: PA14 supernatant (in RPMI with iron, concentrations 1:256 to 1:1024) or PA14ΔpvdD/ΔpchE supernatant at low concentrations (1:256 to 1:1024), combined with VCZ (µM); combined effects against 10AF forming biofilm. Table S4: Bliss Independence Model calculation: pyoverdine (µM) and VCZ (µM) combination effects against 10AF forming biofilm. Table S5: Bliss Independence Model calculation: pyochelin (µM) and VCZ (µM) combination effects against 10AF forming biofilm. Table S6: Bliss Independence Model calculation: upper part: PA14 supernatant (in RPMI with iron concentrations 1:4 to 1:16), lower part: pyocyanin (µM). Combination effects with VCZ (µM) against 10AF forming biofilm.

## References

[1] Riordan J.R., Rommens J.M., Kerem B., Alon N., Rozmahel R., Grzelczak Z., Zielenski J., Lok S., Plavsic N., Chou J.L. (1989). Identification of the cystic fibrosis gene: Cloning and characterization of complementary DNA. Science.

[2] Collins F., Jurivich D., Sistonen L., Kroes R., Morimoto R. (1992). Cystic fibrosis: Molecular biology and therapeutic implications. Science.

[3] Rowe S.M., Miller S., Sorscher E.J. (2005). Cystic fibrosis. N. Engl. J. Med..

[4] King J., Brunel S.F., Warris A. (2016). Aspergillus infections in cystic fibrosis. J. Infect..

[5] O’Brien S., Fothergill J.L. (2017). The role of multispecies social interactions in shaping *Pseudomonas aeruginosa* pathogenicity in the cystic fibrosis lung. FEMS Microbiol. Lett..

[6] Gentzsch M., Mall M.A. (2018). Ion Channel Modulators in Cystic Fibrosis. Chest.

[7] Baxter C.G., Dunn G., Jones A.M., Webb K., Gore R., Richardson M.D., Denning D.W. (2013). Novel immunologic classification of aspergillosis in adult cystic fibrosis. J. Allergy Clin. Immunol..

[8] Moss R.B. (2015). Fungi in Cystic Fibrosis and Non–Cystic Fibrosis Bronchiectasis. Semin. Respir. Crit. Care Med..

[9] Agarwal R., Chakrabarti A., Shah A., Gupta D., Meis J.F., Guleria R., Moss R., Denning D.W., ABPA complicating asthma ISHAM working group (2013). Allergic bronchopulmonary aspergillosis: Review of literature and proposal of new diagnostic and classification criteria. Clin. Exp. Allergy.

[10] Chishimba L., Niven R.M., Cooley J., Denning D.W. (2012). Voriconazole and Posaconazole Improve Asthma Severity in Allergic Bronchopulmonary Aspergillosis and Severe Asthma with Fungal Sensitization. J. Asthma.

[11] Hogan C., Denning D.W. (2011). Allergic Bronchopulmonary Aspergillosis and Related Allergic Syndromes. Semin. Respir. Crit. Care Med..

[12] Perisson C., Destruys L., Grenet D., Bassinet L., Derelle J., Sermet-Gaudelus I., Thumerelle C., Prevotat A., Rosner V., Clement A. (2017). Omalizumab treatment for allergic bronchopulmonary aspergillosis in young patients with cystic fibrosis. Respir. Med..

[13] Amin R., Dupuis A., Aaron S.D., Ratjen F. (2010). The Effect of Chronic Infection with *Aspergillus fumigatus* on Lung Function and Hospitalization in Patients with Cystic Fibrosis. Chest.

[14] Reece E., Segurado R., Jackson A., McClean S., Renwick J., Greally P. (2017). Co-colonisation with *Aspergillus fumigatus* and *Pseudomonas aeruginosa* is associated with poorer health in cystic fibrosis patients: An Irish registry analysis. BMC Pulm. Med..

[15] Briard B., Bomme P., Lechner B.E., Mislin G.L.A., Lair V., Prévost M.-C., Latgé J.-P., Haas H., Beauvais A. (2015). *Pseudomonas aeruginosa* manipulates redox and iron homeostasis of its microbiota partner *Aspergillus fumigatus* via phenazines. Sci. Rep..

[16] Briard B., Rasoldier V., Bomme P., Elaouad N., Guerreiro C., Chassagne P., Muszkieta L., Latgé J.-P., Mulard L., Beauvais A. (2017). Dirhamnolipids secreted from *Pseudomonas aeruginosa* modify antifungal susceptibility of *Aspergillus fumigatus* by inhibiting β1,3 glucan synthase activity. ISME J..

[17] Nazik H., Choudhary V., Stevens D.A. (2017). Verapamil Inhibits *Aspergillus* Biofilm, but Antagonizes Voriconazole. J. Fungi (Basel).

[18] Elliot R.P. (1958). Some properties of pyoverdine, the water-soluble fluorescent pigment of the pseudomonads. Appl. Microbiol..

[19] Chatterjee P., Sass G., Swietnicki W., Stevens D.A. (2020). Review of Potential Pseudomonas Weaponry, Relevant to the *Pseudomonas–Aspergillus* Interplay, for the Mycology Community. J. Fungi (Basel).

[20] Stites S.W., Walters B., O’Brien-Ladner A.R., Bailey K., Wesselius L.J. (1998). Increased Iron and Ferritin Content of Sputum from Patients with Cystic Fibrosis or Chronic Bronchitis. Chest.

[21] Wang J., Lory S., Ramphal R., Jin S. (1996). Isolation and characterization of *Pseudomonas aeruginosa* genes inducible by respiratory mucus derived from cystic fibrosis patients. Mol. Microbiol..

[22] Nazik H., Sass G., Ansari S.R., Ertekin R., Haas H., Déziel E., Stevens D.A. (2020). Novel intermicrobial molecular interaction: *Pseudomonas aeruginosa* Quinolone Signal (PQS) modulates *Aspergillus fumigatus* response to iron. Microbiology.

[23] Sass G., Ansari S.R., Dietl A.-M., Déziel E., Haas H., Stevens D.A. (2019). Intermicrobial interaction: *Aspergillus fumigatus* siderophores protect against competition by *Pseudomonas aeruginosa*. PLoS ONE.

[24] Bargon J., Dauletbaev N., Köhler B., Wolf M., Posselt H.-G., Wagner T.O.F. (1999). Prophylactic antibiotic therapy is associated with an increased prevalence of *Aspergillus* colonization in adult cystic fibrosis patients. Respir. Med..

[25] Denning D.W., Park S., Lass-Florl C., Fraczek M.G., Kirwan M., Gore R., Smith J., Bueid A., Moore C.B., Bowyer P. (2011). High-frequency Triazole Resistance Found In Nonculturable *Aspergillus fumigatus* from Lungs of Patients with Chronic Fungal Disease. Clin. Infect. Dis..

[26] Morio F., Aubin G.G., Danner-Boucher I., Haloun A., Sacchetto E., Garcia-Hermoso D., Bretagne S., Miegeville M., Le Pape P. (2012). High prevalence of triazole resistance in *Aspergillus fumigatus*, especially mediated by TR/L98H, in a French cohort of patients with cystic fibrosis. J. Antimicrob. Chemother..

[27] Mortensen K.L., Jensen R.H., Johansen H.K., Skov M., Pressler T., Howard S.J., Leatherbarrow H., Mellado E., Arendrup M.C. (2011). *Aspergillus* Species and Other Molds in Respiratory Samples from Patients with Cystic Fibrosis: A Laboratory-Based Study with Focus on *Aspergillus fumigatus* Azole Resistance. J. Clin. Microbiol..

[28] Burgel P.-R., Baixench M.-T., Amsellem M., Audureau E., Chapron J., Kanaan R., Honoré I., Dupouy-Camet J., Dusser D., Klaassen C.H. (2012). High Prevalence of Azole-Resistant *Aspergillus fumigatus* in Adults with Cystic Fibrosis Exposed to Itraconazole. Antimicrob. Agents Chemother..

[29] Stevens D.A., Moss R.B., Hernandez C., Clemons K.V., Martinez M. (2016). Effect of Media Modified To Mimic Cystic Fibrosis Sputum on the Susceptibility of *Aspergillus fumigatus*, and the Frequency of Resistance at One Center. Antimicrob. Agents Chemother..

[30] Denning D.W., Clemons K.V., Hanson L.H., Stevens D.A., Morrison D.C., Silverstein R., Bright S.W., Chen T.-Y., Flebbe L.M., Lei M.-G. (1990). Restriction Endonuclease Analysis of Total Cellular DNA of *Aspergillus fumigatus* Isolates of Geographically and Epidemiologically Diverse Origin. J. Infect. Dis..

[31] Denning D.W., Stevens D.A. (1991). Efficacy of cilofungin alone and in combination with amphotericin B in a murine model of disseminated aspergillosis. Antimicrob. Agents Chemother..

[32] O’Toole G.A., Kolter R. (1998). Flagellar and twitching motility are necessary for *Pseudomonas aeruginosa* biofilm development. Mol. Microbiol..

[33] Lee D.G., Urbach J.M., Wu G., Liberati N.T., Feinbaum R.L., Miyata S., Diggins L.T., He J., Saucier M., Déziel E. (2006). Genomic analysis reveals that *Pseudomonas aeruginosa* virulence is combinatorial. Genome Biol..

[34] Fischer S., Klockgether J., Losada P.M., Chouvarine P., Cramer N., Davenport C.F., Dethlefsen S., Dorda M., Goesmann A., Hilker R. (2016). Intraclonal genome diversity of the major *Pseudomonas aeruginosa* clones C and PA14. Environ. Microbiol. Rep..

[35] Stover C.K., Pham X.Q., Erwin A.L., Mizoguchi S.D., Warrener P., Hickey M.J., Brinkman F.S.L., Hufnagle W.O., Kowalik D.J., Lagrou M. (2000). Complete genome sequence of *Pseudomonas aeruginosa* PAO1, an opportunistic pathogen. Nature.

[36] Ferreira J.A.G., Penner J.C., Moss R.B., Haagensen J.A.J., Clemons K.V., Spormann A.M., Nazik H., Cohen K., Banaei N., Carolino E. (2015). Inhibition of *Aspergillus fumigatus* and Its Biofilm by *Pseudomonas aeruginosa* Is Dependent on the Source, Phenotype and Growth Conditions of the Bacterium. PLoS ONE.

[37] Scudiero D.A., Shoemaker R.H., Paull K.D., Monks A., Tierney S., Nofziger T.H., Currens M.J., Seniff D., Boyd M.R. (1988). Evaluation of a soluble tetrazolium/formazan assay for cell growth and drug sensitivity in culture using human and other tumor cell lines. Cancer Res..

[38] Eliopoulos G.M., Eliopoulos C.T. (1988). Antibiotic combinations: Should they be tested?. Clin. Microbiol. Rev..

[39] Zhao W., Sachsenmeier K., Zhang L., Sult E., Hollingsworth R.E., Yang H. (2014). A New Bliss Independence Model to Analyze Drug Combination Data. J. Biomol. Screen..

