# Supplementary material for: Pseudomonas aeruginosa Virulence Factors Support Voriconazole Effects on Aspergillus fumigatus
Source: Pathogens. 2021 Apr 26;10(5):519. doi: 10.3390/pathogens10050519 (PMC8146861; doi:10.3390/pathogens10050519)
Supplement: Supplementary file 1 [file pathogens-10-00519-s001.zip › pathogens-1170880-supplementary.pdf]

Table S1: Bliss Independence Model calculation: PA14 supernatant and VCZ [µM] combined effects against 10AF forming biofilm:

	Y_a [%]	Y_b [%]	Bliss Independence Model [Y^p_{ab}] [%]	[Y^o_{ab}] [%]	Outcome
**VCZ 0. 25 + PA14 sup 1:256**	85	70	96	96	I
**VCZ 0. 25 + PA14 sup 1:512**	85	22	88	96	S
**VCZ 0. 25 + PA14 sup 1:1024**	85	7	86	93	S
**VCZ 0.125 + PA14 sup 1:256**	44	70	83	93	S
**VCZ 0.125 + PA14 sup 1:512**	44	22	56	72	S
**VCZ 0.125 + PA14 sup 1:1024**	44	7	48	73	S
**VCZ 0.063 + PA14 sup 1:256**	20	70	76	73	I
**VCZ 0.063 + PA14 sup 1:512**	20	22	38	41	S
**VCZ 0.063 + PA14 sup 1:1024**	20	7	26	35	S

Abbreviations used in the Table: S = Synergy, I = Independence, Y_a = inhibition of fungal metabolism by VCZ, Y_b = inhibition of fungal metabolism by Pa sup, Y^o_{ab} = observed combined antifungal effect, Y^p_{ab} = predicted combined antifungal effect.

Table S2: Bliss Independence Model calculation: PA14 supernatant and VCZ [µM] combined effects against AF13073 forming biofilm and *P. aeruginosa*, PAO1 and Pa10, supernatants, combined with VCZ against 10AF forming biofilm:

	Y_a [%]	Y_b [%]	Bliss Independence Model [Y^p_{ab}] [%]	[Y^o_{ab}] [%]	Outcome
**AF13073 +**					
**VCZ 0.250 + PA14 sup 1:256**	96	76	99	97	I
**VCZ 0.250 + PA14 sup 1:512**	96	28	97	97	I
**VCZ 0.250 + PA14 sup 1:1024**	96	14	97	97	I
**VCZ 0.125 + PA14 sup 1:256**	63	76	91	92	I
**VCZ 0.125 + PA14 sup 1:512**	63	28	73	83	S
**VCZ 0.125 + PA14 sup 1:1024**	63	14	68	80	S
**VCZ 0.063 + PA14 sup 1:256**	38	76	85	88	I
**VCZ 0.063 + PA14 sup 1:512**	38	28	55	72	S
**VCZ 0.063 + PA14 sup 1:1024**	38	14	47	59	S
**VCZ 0.032 + PA14 sup 1:256**	13	75	78	81	I
**VCZ 0.032 + PA14 sup 1:512**	13	28	37	45	S
**VCZ 0.032 + PA14 sup 1:1024**	13	16	27	30	S
**10AF +**					
**VCZ 0.250 + PAO1 sup 1:128**	84	88	98	95	I
**VCZ 0.250 + PAO1 sup 1:256**	84	85	98	95	I
**VCZ 0.250 + PAO1 sup 1:512**	84	37	90	94	I
**VCZ 0.250 + PAO1 sup 1:1024**	84	14	86	92	S
**VCZ 0.125 + PAO1 sup 1:128**	50	88	94	94	I
**VCZ 0.125 + PAO1 sup 1:256**	50	85	93	92	I

**VCZ 0.125 + PAO1 sup 1:512**	50	37	69	74	S
**VCZ 0.125 + PAO1 sup 1:1024**	50	14	57	68	S
**VCZ 0.063 + PAO1 sup 1:128**	18	88	90	90	I
**VCZ 0.063 + PAO1 sup 1:256**	18	85	88	87	I
**VCZ 0.063 + PAO1 sup 1:512**	18	37	48	53	S
**VCZ 0.063 + PAO1 sup 1:1024**	18	14	29	36	S
**10AF +**					
**VCZ 0.250 + Pa10 Sup 1:64**	88	86	98	96	I
**VCZ 0.250 + Pa10 Sup 1:128**	88	45	93	96	I
**VCZ 0.250 + Pa10 Sup 1:256**	88	19	90	96	S
**VCZ 0.125 + Pa10 Sup 1:64**	55	86	94	95	I
**VCZ 0.125 + Pa10 Sup 1:128**	55	45	75	77	I
**VCZ 0.125 + Pa10 Sup 1:256**	55	19	64	75	S
**VCZ 0.063 + Pa10 Sup 1:64**	27	86	90	91	I
**VCZ 0.063 + Pa10 Sup 1:128**	27	45	60	63	I
**VCZ 0.063 + Pa10 Sup 1:256**	27	19	41	38	A

Abbreviations used in the Table: S = Synergy, I = Independence, Y_a = inhibition of fungal metabolism by VCZ, Y_b = inhibition of fungal metabolism by Pa sup, Y^o_{ab} = observed combined antifungal effect, Y^p_{ab} = predicted combined antifungal effect.

Table S3: Bliss Independence Model calculation: PA14 supernatant (in RPMI with iron, concentrations 1:256 to 1:1024), or PA14ΔpvdD/ΔpchE supernatant at low concentrations (1:256 to 1:1024), combined with VCZ [µM]; combined effects against 10AF forming biofilm:

	Y_a [%]	Y_b [%]	Bliss Independence Model [Y^p_{ab}] [%]	[Y^o_{ab}] [%]	Outcome
**VCZ 0.250 + PA14 sup (Fe) 1:256**	93	9	94	89	I
**VCZ 0.250 + PA14 sup (Fe) 1:512**	93	11	94	91	I
**VCZ 0.250 + PA14 sup (Fe) 1:1024**	93	6	93	90	I
**VCZ 0.125 + PA14 sup (Fe) 1:256**	62	9	65	57	A
**VCZ 0.125 + PA14 sup (Fe) 1:512**	62	11	66	60	A
**VCZ 0.125 + PA14 sup (Fe) 1:1024**	62	6	64	56	A
**VCZ 0.063 + PA14 sup (Fe) 1:256**	28	9	34	28	A
**VCZ 0.063 + PA14 sup (Fe) 1:512**	28	11	36	26	A
**VCZ 0.063 + PA14 sup (Fe) 1:1024**	28	6	32	31	I
**VCZ 0.250 + PA14ΔpvdD/ΔpchE sup 1:256**	85	9	86	81	A
**VCZ 0.250 + PA14ΔpvdD/ΔpchE sup 1:512**	85	14	87	81	A
**VCZ 0.250 + PA14ΔpvdD/ΔpchE sup 1:1024**	85	11	87	81	A
**VCZ 0.125 + PA14ΔpvdD/ΔpchE sup 1:256**	41	9	46	45	I
**VCZ 0.125 + PA14ΔpvdD/ΔpchE sup 1:512**	41	14	49	45	A
**VCZ 0.125 + PA14ΔpvdD/ΔpchE sup 1:1024**	41	11	47	47	I
**VCZ 0.063 + PA14ΔpvdD/ΔpchE sup 1:256**	17	9	24	25	I
**VCZ 0.063 + PA14ΔpvdD/ΔpchE sup 1:512**	17	14	29	23	A
**VCZ 0.063 + PA14ΔpvdD/ΔpchE sup 1:1024**	17	11	26	21	A

Abbreviations used in the Table: S = Synergy, I = Independence, Y_a = inhibition of fungal metabolism by VCZ, Y_b = inhibition of fungal metabolism by Pa sup, Y^o_{ab} = observed combined antifungal effect, Y^p_{ab} = predicted combined antifungal effect]

Table S4: Bliss Independence Model calculation: Pyoverdine [µM] and VCZ [µM] combination effects against 10AF forming biofilm:

	Y_a [%]	Y_b [%]	Bliss Independence Model [Y^p_{ab}] [%]	[Y^o_{ab}] [%]	Outcome
**VCZ 0.125 + pyoverdine 0.63**	56	99	100	99	I
**VCZ 0.125 + pyoverdine 0.32**	56	77	90	97	S
**VCZ 0.125 + pyoverdine 0.16**	56	32	70	72	I

Abbreviations used in the Table: S = Synergy, I = Independence, Y_a = inhibition of fungal metabolism by VCZ, Y_b = inhibition of fungal metabolism by pyoverdine, Y^o_{ab} = observed combined antifungal effect, Y^p_{ab} = predicted combined antifungal effect.

Table S5: Bliss Independence Model calculation: Pyochelin [µM] and VCZ [µM] combination effects against 10AF forming biofilm

	Y_a [%]	Y_b [%]	Bliss Independence Model [Y^p_{ab}] [%]	[Y^o_{ab}] [%]	Outcome
**VCZ 0.250 + pyochelin 100**	96	73	99	97	I
**VCZ 0.250 + pyochelin 50**	96	73	99	97	I
**VCZ 0.250 + pyochelin 25**	96	78	99	97	I
**VCZ 0.125 + pyochelin 100**	65	20	72	69	I
**VCZ 0.125 + pyochelin 50**	65	20	72	75	I
**VCZ 0.125 + pyochelin 25**	65	2	66	78	S
**VCZ 0.063 + pyochelin 100**	43	73	85	86	I
**VCZ 0.063 + pyochelin 50**	43	73	85	90	S
**VCZ 0.063 + pyochelin 25**	43	78	87	94	S

Abbreviations used in the Table: S = Synergy, I = Independence, Y_a = inhibition of fungal metabolism by VCZ, Y_b = inhibition of fungal metabolism by pyochelin, Y^o_{ab} = observed combined antifungal effect, Y^p_{ab} = predicted combined antifungal effect.

Table S6: Bliss Independence Model calculation: upper part: PA14 supernatant (in RPMI with iron concentrations 1:4 to 1:16), lower part: pyocyanin [µM]. Combination effects with VCZ [µM] against 10AF forming biofilm.

	Y_a [%]	Y_b [%]	Bliss Independence Model [Y^p_{ab}] [%]	[Y^o_{ab}] [%]	Outcome
**VCZ 0.250 + PA14 sup (Fe) 1:4**	98	48	99	100	I
**VCZ 0.250 + PA14 sup (Fe) 1:8**	98	42	99	100	I
**VCZ 0.250 + PA14 sup (Fe) 1:16**	98	16	98	100	I
**VCZ 0.125 + PA14 sup (Fe) 1:4**	67	48	83	93	S
**VCZ 0.125 + PA14 sup (Fe) 1:8**	67	42	81	96	S
**VCZ 0.125 + PA14 sup (Fe) 1:16**	67	16	72	74	I
**VCZ 0.063 + PA14 sup (Fe) 1:4**	44	48	71	76	S
**VCZ 0.063 + PA14 sup (Fe) 1:8**	44	42	68	77	S
**VCZ 0.063 + PA14 sup (Fe) 1:16**	44	16	53	55	I
**VCZ 0.125 + pyocyanin 1000**	67	76	92	94	I
**VCZ 0.125 + pyocyanin 500**	67	53	84	85	I
**VCZ 0.125 + pyocyanin 250**	67	36	79	68	A

Abbreviations used in the Table: S = Synergy, I = Independence, Y_a = inhibition of fungal metabolism by respective VCZ, Y_b = inhibition of fungal metabolism by Pa sup or pyocyanin, Y^o_{ab} = observed combined antifungal effect, Y^p_{ab} = predicted combined antifungal effect]
